# MWCNT Localization and Electrical Percolation in Thin Films of Semifluorinated PMMA Block Copolymers

**DOI:** 10.3390/polym17091271

**Published:** 2025-05-06

**Authors:** Ulrike Staudinger, Andreas Janke, Frank Simon, Lothar Jakisch, Eva Bittrich, Petr Formanek, Lukas Mielke, Hendrik Schlicke, Qiong Li, Kathrin Eckstein, Doris Pospiech

**Affiliations:** Leibniz-Institut für Polymerforschung Dresden e.V., Hohe Str. 6, 01069 Dresden, Germanyfrsimon@ipfdd.de (F.S.); bittrich-eva@ipfdd.de (E.B.); formanek@ipfdd.de (P.F.); mielke-lukas@ipfdd.de (L.M.); schlicke@ipfdd.de (H.S.); li-qiong@ipfdd.de (Q.L.);

**Keywords:** diblock copolymer, carbon nanotubes, CNT localization, morphology, filler-matrix interaction, thin films, electrical conductivity, atomic force microscopy

## Abstract

Diblock copolymers (BCP) consisting of poly(methyl methacrylate) (PMMA) and poly(1H,1H,2H,2H-perfluorodecyl methacrylate) (PsfMA) blocks are employed as templates for controlled dispersion and localization of multi-walled carbon nanotubes (MWCNT). Short MWCNT are modified with perfluoroalkyl groups to increase the compatibility between MWCNT and the semifluorinated (PsfMA) phase and to promote a defined arrangement of MWCNT in the BCP morphology. Thin BCP and BCP/MWCNT composite films are prepared by dip-coating using tetrahydrofuran as solvent with dispersed MWCNT. Atomic force microscopy, scanning and transmission electron microscopy reveal a strong tendency of the BCP to form micelle-like domains consisting of a PMMA shell and a semifluorinated PsfMA core, embedded in a soft phase, containing also semifluorinated blocks. MWCNT preferentially localized in the embedding phase outside the micelles. Perfluoroalkyl-modification leads to significant improvement in the dispersion of MWCNT, both in the polymer solution and the resulting nanocomposite film due to increased interaction of MWCNT with the semifluorinated side chains in the soft phase outside the micelle domains. As a result, reliable electrical conductivity is observed in contrast to films with non-modified MWCNT. Thus, well-dispersed, modified MWCNT provide a defined electrical conduction path at the micrometer level, which is interesting for applications in electronics and vapor sensing.

## 1. Introduction

The development and application of novel hybrid materials, comprising inorganic nanomaterials such as carbon-based fillers embedded in a polymer matrix and enabling efficient solution-based processing, are gaining increasing attention due to their unique electrical properties and potential for future use in sensing and related technologies. These materials can be used, for example, in applications for biosensor technology [1], as transparent electrodes for solar cells, electromagnetic interference shielding and energy storage or for thin film transistors and in optoelectronic devices [2,3].

The use of carbon nanotubes (CNT) as nanofillers with high aspect ratio and exceptional mechanical, thermal and electrical properties offers the possibility to tune the electrical conductivity of CNT composites with very low filler contents because the electrical percolation threshold can be significantly reduced with good dispersion of the CNT compared to spherical particles [4,5].

For this reason, multiwalled (MW) CNT were chosen as electrical conducting nanofillers for this study to prepare thin, electrically conductive polymer films applicable in nanoelectronics.

As base polymer, poly(methyl methacrylate) (PMMA) was selected due to its excellent film-forming properties necessary to prepare thin films with thicknesses of a few nanometers [6,7,8].

Most of the studies reporting PMMA/MWCNT nanocomposites used melt-mixing to prepare the samples [9], only some prepared films from solution [10,11]. The electrical percolation thresholds in both cases were rather low (between 0.5 and 0.75 wt.% MWCNT for melt-mixed samples and at between 0.07 and 0.2 wt.-% MWCNT for solution-mixed samples), with good distribution within in the PMMA matrix. With these materials in a thickness of several micrometers, the applicability of PMMA/CNT conducting films for gas sensing was demonstrated [10].

However, the CNT distribution has to be even better to achieve thin, homogeneously conductive films with structures in the nanometer scale. Therefore, the use of block copolymers (BCP) was considered for this study to employ the nanostructure formed by the phase separation of the BCP to arrange the CNT in a regular manner.

BCP consist of two or more blocks of chemically different polymers that are covalently linked together. Thermodynamically immiscible polymer blocks self-organize into ordered, microphase-separated structures at the nanoscale due to phase separation, leading to the formation of exceptional morphologies [12,13]. Depending on the degree of polymerization of the blocks, the volume fractions of the individual blocks, and the Flory-Huggins interaction parameter, stable lamellar, cylindrical, spherical and other three-dimensional morphologies can be tailored in a controlled way [14,15,16,17,18]. Due to their defined nanostructures, BCP are very suitable as templates for the dispersion and localization of nanoscale fillers such as 0-dimensional spherical particles (e.g., gold or silver nanoparticles, quantum dots) [19,20,21,22,23], one-dimensional tubes (like CdSe rods [24], CNT [25,26,27,28,29,30,31]) or two-dimensional plate-shaped particles (e.g., graphene, silicate) [23,32,33].

There are several studies, which used the chemical modification of CNT to selectively arrange the nanoobjects in one phase of BCP [14,15,16,17,18,19,20]. We have summarized these studies in detail in our previous publications [27,28,29,30]. To be embedded in the nanodomains of the BCP, at least one of the dimensions of the nanofiller must be in the order of the BCP domain size (typically 5–100 nm) [34,35]. The well-defined arrangement of nanoparticles in block copolymer nanostructures enables the development of functional nanocomposites with specific mechanical, optical, electrical, photovoltaic and barrier characteristics [35].

Another approach is to use BCP as nanotemplates for selective deposition of metal or metal oxide catalysts to induce controlled growth of nanotubes in hierarchically organized vertical arrays [36,37].

In our studies on polystyrene (PS)-polybutadiene (PB)-based starblock copolymer/MWCNT nanocomposites, we demonstrated a significant influence of CNT modification on the dispersion and localization behavior of CNT in the nanostructured phase morphology of the BCP using a solution mixing process [38,39]. In particular, the controlled shortening of MWCNT had a positive effect on the dispersibility of CNT and resulted in preferential localization of CNT in the soft PB-rich phase of the BCP. The electrical percolation threshold was slightly increased by shortening compared to composites with untreated CNT [39]. In a linear PS-*b*-poly(S-*co*-B)-*b*-PS triblock copolymer, ball-milled MWCNT with an average length (D50) of ~500 nm were dispersed by melt blending. TEM images suggested affinity or partial localization of CNT in the soft PB-rich phase [40].

In addition, Santos et al. [41] found that the incorporation of CNT influenced the self-assembly of styrene-*b*-butadiene-*b*-styrene block copolymers. Furthermore, the electrical percolation threshold can be tuned by the variation in the block composition as investigated by Topcu et al. [42]. An increase in the styrene fraction of poly(styrene-*b*-2-ethylhexyl acrylate) BCP led to a decrease in the required MWCNT fraction to obtain conductive composites.

In the present study, we used the nanostructured morphology of semifluorinated PMMA block copolymers for the controlled incorporation of short, chemically modified MWCNT to generate homogeneous, defined long-range electrical percolation within the BCP matrix. Semifluorinated methacrylate copolymers with random chain structure have already been demonstrated to yield a lower percolation threshold with MWCNT than non-modified PMMA [43].

A further goal was to find out whether or not the MWCNT would arrange within one phase of the BCP (i.e., whether or not the BCP can form a template for the MWCNT arrangement). The MWCNT arrangement was examined by means of scanning electron microscopy (SEM), transmission electron microscopy (TEM) and atomic force microscopy (AFM), while the electrical properties were analyzed by using conductive AFM (cAFM), electrical force gradient microscopy (EFM) and electrical conductivity measurements of thin films deposited onto lithographically fabricated electrode microstructures.

## 2. Materials and Methods

### 2.1. Materials

The synthesis of the diblock copolymers consisting of a PMMA and a poly(1H,1H,2H,2H-perfluorodecyl methacrylate) (PsfMA) block was performed by anionic polymerization and has been described elsewhere [44]. A summary of the chemical characterization of the PMMA-*b*-PsfMA diblock copolymers used in this study is given in Table 1. Shortened multiwalled carbon nanotubes (sMWCNT) were provided by Nanocyl SA (Sambreville, Belgium). Shortening of MWCNT grade NC7000, with an average diameter of ~9.5 nm and an average length of ~0.5 µm [45] was performed by grinding in a mixer mill for 10 h to about 35% of their original length as described elsewhere [46]. 1H,1H,2H,2H-Perfluorododecan-1-ol (97%, abcr GmbH, Karlsruhe, Germany) was used as received. Benzoyl peroxide was recrystallized from methanol. All other chemicals and solvents were obtained from Aldrich and used without further purification.

### 2.2. Modification of sMWCNT

Two approaches to modify the short sMWCNT were used. Both modification procedures resulted in the same chemical structure of the functional groups.

In the first approach, perfluoroalkyl-modified sMWCNT were synthesized according to the procedure described by C. E. Hamilton et al. [49] using 1-iodo-1H,1H,2H,2H-perfluorodecane.

sMWCNT (40 mg) and 1-iodo-1H,1H,2H,2H-perfluorodecane (5.40 g, 9.41 mmol) were reacted in *o*-dichlorobenzene (60 mL) in presence of recrystallized benzoyl peroxide (0.80 g, 3.30 mmol) for 24 h at 80 °C. After cooling to room temperature, chloroform (60 mL) was added. The solid was filtered through a 0.2 µm hydrophobic poly(tetrafluoroethylene) membrane (FluoporeTM Membrane Filters, Merck Millipore Ltd., Cork, Ireland) and washed with chloroform. After that, the solid was redispersed three times for 10 min in chloroform (100 mL) using an ultrasonic bath. After drying under reduced pressure at 50 °C for 8 h, perfluoroalkyl-modified sMWCNT (32 mg) were obtained (sMWCNT_pf1).

According to the procedure with NC7000 described in our previous report [38], in the second approach, the sMWCNT were slightly oxidized with 60% aqueous HNO_3_ to sMWCNT-COOH followed by the reaction of the resulting carboxylic acid groups with thionyl chloride to the respective acid chloride sMWCNT-COCl.

After that, sMWCNT-COCl (1.0 g) were reacted with 1H,1H,2H,2H-perfluorododecan-1-ol (1.00 g, 1.77 mmol) and triethylamine (1.00 g, 10 mmol) in dry tetrahydrofuran (THF, 70 mL) at room temperature under stirring for 12 h. After filtration through a 0.2 µm hydrophobic poly(tetrafluoroethylene) membrane, the remaining solid was washed successively with THF, ethanol, and chloroform.

In order to remove traces of unreacted perfluorododecan-1-ol, the solid was heated in chloroform (150 mL) under reflux for 2 h, filtered through a 0.2 µm poly(tetrafluoroethylene) membrane, and washed with chloroform. After drying under reduced pressure at 50 °C for 8 h, perfluoroalkyl-modified carbon nanotubes (1.0 g) were obtained (sMWCNT_pf2).

### 2.3. Thermogravimetric Analysis

The pristine MWCNT NC7000, the shortened sMWCNT, and the perfluoroalkyl-modified sMWCNT were analyzed by thermogravimetric analysis (TGA) in nitrogen atmosphere using a TGA Q5000 (TA-Instruments, New Castle, DE, USA). The constant heating rate up to 800 °C was 10.0 K/min.

### 2.4. Characterization of CNT Dispersion in Solvents

To assess the dispersibility of the functionalized MWCNT in solvents, 0.5 mg each of original NC7000, ground sMWCNT and functionalized sMWCNTs_pf2 were dispersed in THF (10 mL) with the aid of an UP400S ultrasonic processor using sonotrode H3 (both from Hielscher Ultrasonics GmbH, Teltow, Germany) at an amplitude of 50% for 2 min. The MWCNT dispersions were photographed immediately after dispersing as well as after 1 h, 2 h and 24 h. The quality and stability of the dispersions was assessed by visual observation.

### 2.5. Preparation of Thin Polymer and Nanocomposite Films

Polymer solutions were prepared by dissolving 1 wt% (0.089 g) of the BCP in THF (10 mL) by magnetic stirring for 1 h. For the preparation of BCP1/sMWCNT_pf2 composites containing 0.5 wt% of MWCNT, the polymer was dissolved in only 5 mL of solvent and sMWCNT_pf2 were dispersed separately in 5 mL of solvent using the abovementioned ultrasonic processor at an amplitude of 50% for 5 min. Then, the polymer solution was added to the MWCNT dispersion, stirred for another 30 min and ultrasonicated again for 1 min.

Silicon wafers (size 10 mm × 15 mm) served as substrates and were cleaned according to the RCA (Radio Corporation of America) standard as described in our recent publication [39]. To prepare thin films, the silicon wafers were dipped vertically into the polymer solution or into the polymer/MWCNT dispersion, respectively, using a custom-built dip-coater and pulled out immediately, varying the pull-out speed between 0.5 mm s^−1^ and 5 mm s^−1^ to produce different layer thicknesses.

For BCP2/sMWCNT_pf1 and BCP3/sMWCNT_pf1 composites, the MWCNT content was varied between 0.5 and 2 wt%. After film preparation for the pure polymers, 0.5 wt% sMWCNT_pf1 were added directly to the polymer stock solution and dispersed by ultrasonic treatment for 5 min. Then, the film preparation was repeated by dip-coating as described above. To increase the total MWCNT amount to 1 wt%, a further portion of 0.5 wt% sMWCNT_pf1 were added to the dispersion and the film preparation was performed. The procedure was repeated until the MWCNT content in the composite film reached 2 wt%. Since the films produced were less than 100 nm thick, the loss of solution due to each dip-coating step is negligible and had no influence on the actual MWCNT concentration.

### 2.6. Evaluation of Film Thickness

Optical properties and thickness of thin BCP films with and without MWCNT were analyzed by spectroscopic ellipsometry (alpha-SE, J. A. Woollam Co. Inc., Lincoln, NE, USA). The ellipsometric data were fitted to a box model of silicon substrate, silicon dioxide, and polymer film [50]. The optical dispersion of silicon and silicon dioxide was taken from the database (CompleteEASE, J. A. Woollam Co. Inc., Lincoln, NE, USA), while the optical constants of the polymer layer were modeled by a Cauchy function (n(λ) = A + B/λ^2^, k = 0) [51]. The MWCNT content in the films was small enough (≤2%) that also polymer films with MWCNT could be modeled with a Cauchy function.

### 2.7. Surface Spectroscopy of MWCNTs and BCP/MWCNT Layers

X-ray photoelectron spectroscopy (XPS) was employed to study the surface chemistry of the MWCNT samples as well as their composites with BCP. The XPS method is a surface sensitive technique for qualitative and quantitative elemental analyses of the topmost surface layer (information depth max. 8 nm). All XPS spectra were recorded with an Axis Ultra photoelectron spectrometer (Kratos Analytical, Manchester, UK) equipped with a monochromatic Al Kα (h·ν = 1486.6 eV) X-ray source of 300 W at 15 kV. The kinetic energy of photoelectrons was determined with a hemispheric analyzer set to pass energy of 160 eV for wide-scan spectra and 20 eV for high-resolution spectra. For XPS analyses, the MWCNT samples were prepared as dense particle films onto a double-sided adhesive tape (Scotch, 3 M, Maplewood, MN, USA) mounted on the spectrometer sample holder. During all measurements, electrostatic charging of the sample was avoided by means of a low-energy electron source working in combination with a magnetic immersion lens. Later, all recorded peaks were shifted by the value necessary to set the component peaks for saturated hydrocarbon in the C 1s spectra to 285.00 eV. Quantitative elemental compositions were determined from peak areas using experimentally determined sensitivity factors and the spectrometer transmission function. Spectrum backgrounds were subtracted according to Shirley [52]. The high-resolution spectra were deconvoluted by means of the Kratos spectra deconvolution software Vision Processing, version 2.2.9 (Kratos Analytical, Manchester, UK). Free parameters of component peaks were their binding energy (BE), height, full width at half maximum and the Gaussian–Lorentzian ratio.

### 2.8. Morphological Characterization

To characterize the BCP morphology and the MWCNT dispersion, atomic force microscopy (AFM) was performed. The AFM measurements were taken in the tapping mode by a Dimension FASTSCAN (Bruker Cooperation, Billerica, MA, USA). Silicon-SPM-sensors SCANASYST-FLUID+ (Bruker Cooperation, Billerica, MA, USA) with spring constant of nominally 0.47 N m^−1^ and resonance frequency of nominally 150 kHz, and a tip radius of 2 nm were used. Height images (surface morphology) and phase images were taken simultaneously. According to Magonov et al. [53], the scan conditions (free amplitude > 100 nm, set-point amplitude ratio 0.8) were chosen in order to obtain stiffness contrast in the phase image. That means, larger phase angles refer to stiffer features in the phase image than smaller phase angles.

The software NanoScope Analysis 2.0 (Bruker Cooperation, Billerica, MA, USA) was used to estimate the mean MWCNT particle diameter. The mean distance between domains was calculated by analyzing the 2D-power spectral density (2D-PSD).

For a comprehensive characterization of the BCP morphology as well as MWCNT dispersion and localization, additional scanning electron microscopy (SEM) and transmission electron microscopy (TEM) investigations were carried out. Thin films dip-coated on silicon wafers were inspected in NEON40 scanning electron microscope (Zeiss Microscopy Deutschland GmbH, Oberkochen, Germany) operated at 1 kV to 3 kV. A secondary electron detector was used for topography contrast, energy filtered back-scattered detector was used for material contrast, in-lens electron detector was used for the best contrast of CNT. Specimens for TEM were prepared in two ways. In the first way, solution of BCP and CNT in THF was spread on a water surface and the resulting film was collected on a TEM grid. In the second way, films dip-coated on silicon wafer were coated with ca. 10 nm carbon film (SCD500 coater, Leica Microsystems GmbH, Wetzlar, Germany), flotated on a water film, and collected on TEM grid. The TEM images were recorded using Libra120 transmission electron microscope (Zeiss Microscopy Deutschland GmbH, Oberkochen, Germany) operated at 120 kV. To further verify the BCP phases, elemental maps were acquired by energy-filtered TEM using 3-windows method [54].

### 2.9. Electrical Characterization

AFM was also used to investigate the electrical characteristics of the nanocomposite films. Conductive atomic force microscopy (cAFM) investigations were carried out in contact mode using a Peak Force TUNA application module (Bruker Cooperation, Billerica, MA, USA) for measurement of the contact current. To ensure an electrical contact, silver paste is applied directly onto the conductive thin film. This setup allows the current to flow laterally through the film towards the AFM tip. The chosen sensitivity was 1 nA V^−1^ and a voltage of 400 mV was applied during the scan. Ir/Pt-coated silicon-SPM-sensors SCM-PIT-V2 (Bruker Cooperation, Billerica, MA, USA) with spring constant of nominal 3 N m^−1^ was used, the tip radius was below 25 nm.

Electrical force gradient microscopy (EFM) was performed in tapping mode/lift mode with the SCM-PIT-V2 sensor. The main scan line without applied voltages gives the topography, the second scan line at the same position with a distance (lift height) of 100 nm to the sample surface and applied voltage of −5 V to the tip and +5 V to the sample shows attractive interaction in the phase image due to electrostatic forces, darker regions mean higher attractive force. The electrical AFM measurements cAFM and EFM were all performed by a Dimension ICON microscope (Bruker Cooperation, Billerica, MA, USA). The samples were contacted by silver paste.

In addition, thin films of BCP/MWCNT composites with and without perfluoroalkyl modification were investigated regarding their charge transport characteristics. Therefore, the materials were deposited onto lithographically fabricated, interdigitated electrodes (with 20 fingers per electrode) by dip-coating. The structures were contacted using an MPI TS50 manual probe system and current-voltage characteristics were recorded using a Keithley 2612B source meter. The applied voltages were swept from −5 to 5 V in 0.1 V steps, and the resulting current was monitored. Measurements were conducted on three film samples (A1–A3, 7 individual electrode pairs) from BCP2/modified sMWCNT_pf1 and two film samples (B1–B2, 13 individual electrode pairs) from BCP2/non-modified sMWCNT.

The conductivity was calculated according to Equation (1):

(1) $$\sigma =\frac{G\cdot l}{w\cdot t}$$

Here, G denotes the conductance derived from fits of slope functions to the current-voltage data in the range between −1 and 1 V, l is the channel length (distance between the electrode fingers, which was 10 µm), w is the effective channel width (total overlap width of the interdigitated electrode fingers, *w* = 17.16 mm) and t is the mean thickness of the films measured via topographic AFM. An overview about the determined thicknesses is given in the Supplementary Materials (Table S2 and Figure S1).

## 3. Results

### 3.1. Characterization of the Modified MWCNT

**Figure 1:**
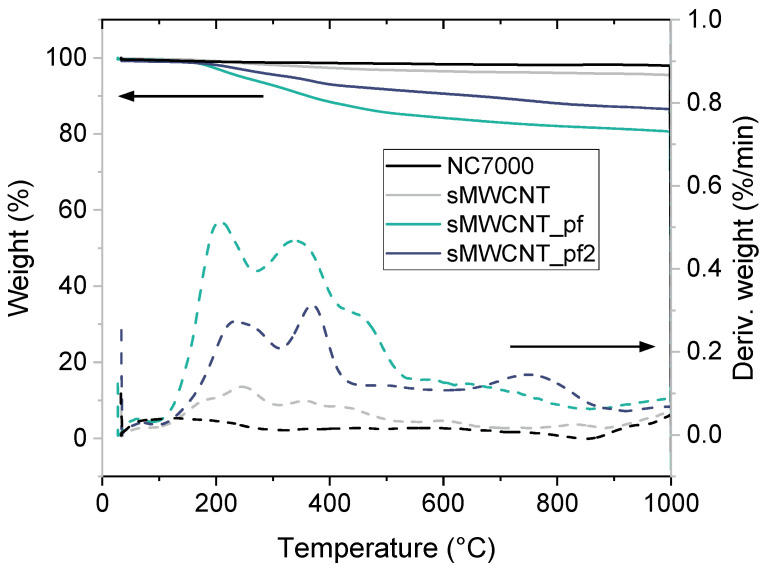
Thermal decomposition of MWCNT NC7000, ground sMWCNT and perfluoroalkyl-modified sMWCNT_pf1 and sMWCNT_pf2, obtained by TGA.

Successful perfluoroalkyl-modification of the sMWCNT was indicated by TGA (Figure 1) and XPS measurements. TGA records showed that after annealing to 1000 °C unmodified MWCNT (NC7000) exhibited a weight loss of only ~2 wt%. Shortened sMWCNT showed a higher weight loss of ~5 wt%, which is probably due to the decomposition of oxidized moieties formed during the grinding process [55]. Modified sMWCNT_pf1 had a significantly increased weight loss of ~20 wt%. sMWCNT_pf2 exhibited a weight loss of ~13 wt%. These results indicated a more effective modification of sMWCNT_pf1 due to more pronounced decomposition of perfluoroalkyl chains compared to MWCNT_pf2. Since the quantity available of sMWCNT_pf1 was very low, these MWCNT were only used to prepare the composites with BCP2 and BCP3. Composites with BCP1 were prepared using sMWCNT_pf2.

**Figure 2:**
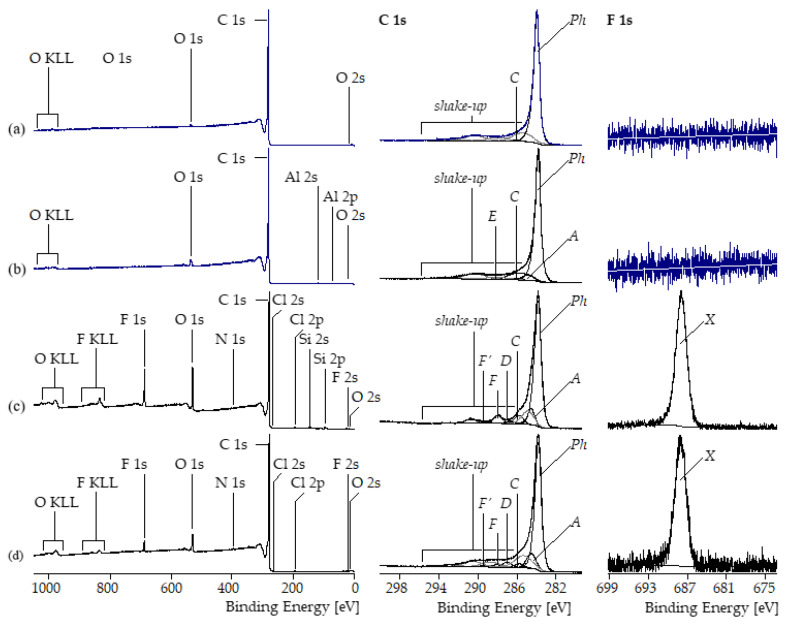
Wide-scan, C 1s and F 1s high-resolution spectra recorded from (**a**) untreated MWCNT NC7000, (**b**) sMWCNT ground for 10 h, (**c**) sMWCNT_pf1, and (**d**) sMWCNT_pf2 samples.

The findings of the TGA studies were confirmed by XPS spectra recorded from the differently treated MWCNT samples. Unmodified MWCNT NC7000 contained only traces of oxygen ([O]:[C] = 0.007, Figure 2a). Grinding for 10 h increased the relative oxygen content of sMWCNT more than fourfold to [O]:[C] = 0.031 (Figure 2b). The appearance of the F 1s photoelectron peaks at 688.42 eV and the F KLL *Auger* series indicate that the fluorination reactions were successfully performed (Figure 2c,d). The two synthetic routes of fluorination led to significantly different fluorine contents on the MWCNT surfaces: [F]:[C]|_MWCNT_pf1_ = 0.059 vs. [F]:[C]|_MWCNT_pf2_ = 0.016. The unimodal distributions of the photoelectrons in the high-resolution F 1s spectra (component peak *X* at 688.5 eV in Figure 2c,d, right column) show that all fluorine atoms were covalently bound to MWCNT surfaces. As a further result of the fluorination reactions, the relative oxygen contents also increased significantly to [O]:[C] = 0.121 for the sMWCNT_pf1 sample and [O]:[C] = 0.058 for the sMWCNT_pf2 sample. The shape of the high-resolution C 1s spectra is—independently of the type of surface modification—characteristic of carbon-containing materials mainly composed of graphite-like lattices (Figure 2, middle column). Photoelectrons escaped from the sp^2^-hybridized carbon atoms in their electronic ground state contributed to the main component peaks *Ph* observed at 284 eV. The emission of photoelectrons from electronically excited states caused intense *shake-up* peaks observed on the high-energy side of the C 1s peaks. The high number of excited states also led to the tailing of the C 1s peaks on their higher energetic sides. The tribomechanical and chemical modifications affected the intensities and the positions of the *shake-up* peaks only slightly. Subtracting the areas of the *shake-up* peaks from the C 1s peak areas, the remaining areas were deconvoluted and assigned to the binding states of the functionalized carbon atoms. After grinding the MWCNT sample, both sp^2^- (component peak *Ph*) and sp^3^-hybridized carbon atoms (component peak *A* at 285.00 eV) were detected. The presence of such saturated hydrocarbons on the surface of sMWCNT is likely due to contamination during the grinding process. Traces of phenolic C–OH groups were assigned to component peak *C* (at 286.26 eV) and the carbonyl carbon atoms of carboxylic acid groups (HO–C=O) were labeled *E* (at 288.21 eV). Component peaks of remarkable intensities attributable to quinone-like bonded carbon atoms (C=O) appeared in the C 1s spectra recorded from the fluorinated MWCNT samples at 287.61 eV. Obviously, the surface fluorination reactions were accompanied by a partial oxidation of the MWCNT surfaces. In addition to the functional surface groups discussed above, the fluorinated samples showed component peaks *F* of considerable intensities at 288.53 eV resulting from difluoromethylene groups (–CF_2_) and component peaks *F’* at 289.57 eV resulting from trifluoromethyl groups (–CF_3_). Photoelectrons from the carbonyl carbon atoms of the carboxylic ester groups formed during the esterification reactions were not separated from the component peaks *F*. The perfluorinated alkyl chains of all substances used as starting materials were composed of difluoro methylene (-CF2-) or trifluoro methyl (-CF3) groups. The carbon atoms of these groups were sp3-hybridized. In the case of the sMWCNT_pf2 samples that were synthesized by an esterification reaction a conversion from the sp^3^ to sp^2^ hybridization state can be excluded. Since the reaction of 1-iodo-1H,1H,2H,2H-perfluorodecane with the ground MWCNTs was radically controlled an abstraction of fluorine radicals (F·) during the reaction cannot be excluded. Those eliminations of F·led to the formation of F-C=C-F double bonds in which the carbon atoms were sp2-hybridized. In our opinion, this potentially possible side reaction is of minor importance. We have no spectroscopic evidence that such bonds were formed or present. In summary, the XPS results as well as the TGA measurements proved successful fluorination of the sMWCNT.

### 3.2. Dispersability of the CNT in THF

**Figure 3:**
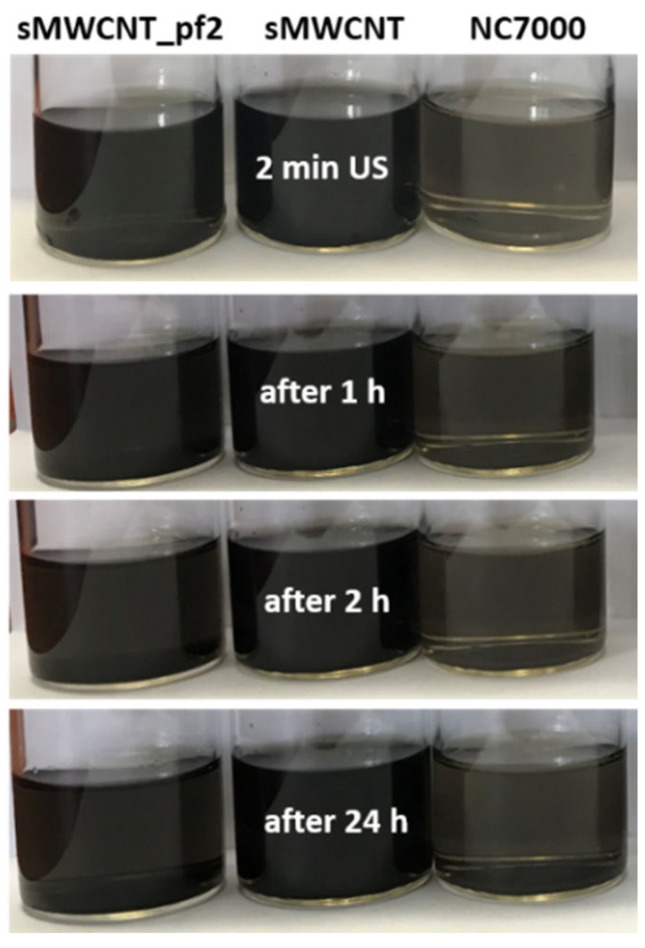
Dispersions of MWCNT NC7000, ground sMWCNT and perfluoroalkyl-modified sMWCNT_pf2 in THF after 2 min of ultrasonication (US) and after rest periods of 1 h, 2 h and 24 h.

MWCNT/THF dispersions with NC7000, ground sMWCNT and perfluoroalkyl-modified sMWCNT_pf2 were assessed and photographed immediately after ultrasonic treatment as well as subsequently 1 h, 2 h and 24 h after ultrasonic treatment to evaluate the quality and stability of the dispersions (Figure 3). In general, all MWCNT grades dispersed well in THF, although residual agglomerates were still detectable in the unmodified MWCNT NC7000 after 2 min of ultrasonic treatment. In comparison, the ground sMWCNT and the modified sMWCNT_pf2 dispersed sufficiently well without agglomerates. sMWCNT exhibited the best stability in THF, as no sedimented MWCNT were observed even after 24 h. In contrast, 2 h after dispersion small amounts of sedimented MWCNT were present in the dispersion with perfluoroalkyl-modified sMWCNT_pf2. From these observations, it can be concluded that THF is suitable to disperse the modified sMWCNT and to keep the dispersion stable for the duration of the film preparation. Perfluoroalkyl groups are very hydrophobic and can improve the dispersion of MWCNTs due to the lower van der Waals attraction and lead to a more homogeneous dispersion of the CNTs. Such a significant increase in the dispersibility of MWCNTs modified with 1,1,2,2,3,3,4,4,5,5,6,6,7,7,8,8,8-heptacafluoro-1-octane was also observed, for example, by Deng et al. when dispersed in THF [56].

### 3.3. Characterization of the BCP Used in This Study

**Figure 4:**
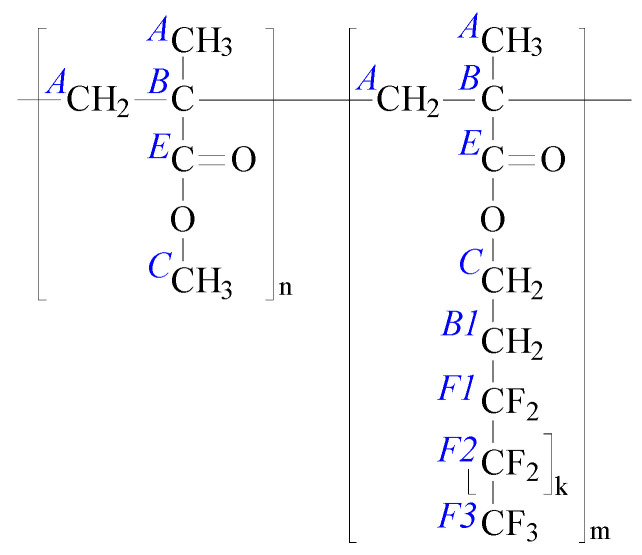
Chemical structure of the PMMA-*b*-PsfMA BCP used in this study (including assignment of the XPS C 1s component peaks of BCP1, see Section 3.4.4; the PMMA block is represented by [C_5_H_8_O_2_]_n_, and the PsfMA block by [C_8+k_H_9_O_2_F_5+2k_]_m_).

The PMMA-*b*- PsfMA diblock copolymers studied here were synthesized by sequential anionic polymerization, as reported earlier [33]. The chemical structure of the PMMA-*b*-PsfMA BCP is depicted in Figure 4.

The BCPs exhibited strong phase separation and ordering into nanostructures below 100 nm as shown by a combination of small-angle X-ray scattering (SAXS) and TEM of the as-synthesized bulk samples as well as of slowly evaporated BCP films [45,48]. SAXS revealed that most of the BCP samples formed a mixture of lamellar and cylindrical morphology. The characterization of the PMMA-b-PsfMA BCP samples selected for the present study is given in Table 1 (see Section 2.1).

Two types of P(MMA-b-sfMA) BCP were chosen: two BCP with a composition of 73/27 mol/mol MMA/sfMA with a bulk morphology characterized by a mixture of cylindrical and lamellar nanostructure (BCP1 and BCP2), with calculated periodic distances in the cylindrical morphology of 41 nm (BCP 1) [48] and 54 nm (BCP 2) [44], assuming hexagonally dense-packed cylinders. The second BCP type exhibits a lamellar bulk morphology (BCP3) with a d-spacing of 45 nm [44,47]. These two types were studied to find out the optimal prerequisite to arrange the sMWCNT within the BCP nanostructure.

As well as the periodic BCP nanostructure, a smaller nanostructure with a d-spacing of about 3 nm was observed originating from the layered arrangement of the semifluorinated side chains [45]. This nanostructure is not relevant for the present study. Relevant, in contrast, is the former observation that PMMA-*b*-PsfMA BCP formed micelles both in diluted THF and chloroform solutions which can be explained by their amphiphilic structure [45]. Thin films prepared by dip-coating (the procedure also employed in this study) may preserve this micellar structure. Former grazing incidence SAXS (GISAXS) measurements on PMMA-*b*-PsfMA thin films with thicknesses up to 40 nm [45] proved phase separation, but did not give clear indications of the type of nanostructure within the thin films because only a single reflection was observable. Therefore, the morphology of these films was examined here again in detail by AFM, SEM and TEM.

### 3.4. Characterization of BCP and BCP/CNT Nanocomposite Films

#### 3.4.1. Film Thickness

**Figure 5:**
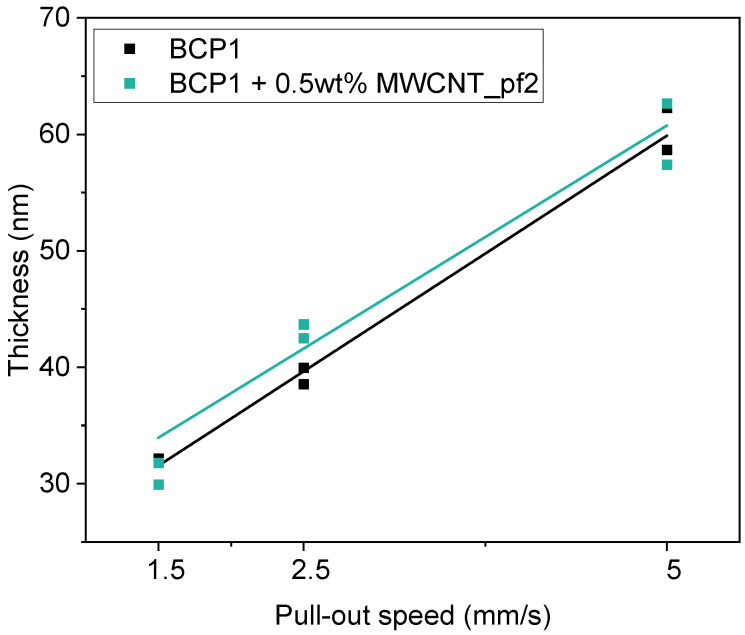
Thickness of dip-coated BCP1 films and BCP1/0.5 wt% sMWCNT_pf2 composite films as a function of pull-out speed.

Thin films of BCP and BCP/sMWCNT composites were obtained by dip-coating on Si substrates by variation in the pull-out speed. The resulting film thicknesses were measured by spectroscopic ellipsometry. A nearly linear dependence of the film thickness on the pull-out speed could be observed, as demonstrated for BCP1 and BCP1/0.5 wt% sMWCNT_pf2 composite films in Figure 5. At low pull-out speed of 0.5 mm s^−1^, the layers were inhomogeneous and showed holes. The Si wafers were not completely wetted by the dispersion. Consequently, for samples prepared with a pull-out speed of 0.5 mm s^−1^, the film thickness could not be determined. Good layer quality and uniform morphology formation could be observed in samples prepared at pull-out speeds of 1.5 and 2.5 mm s^−1^. With increasing pull-out speed, the layer thickness increased continuously. Films prepared at a pull-out speed of 1.5 mm s^−1^ exhibited film thicknesses of ~32 nm (Table 2).

The measured thicknesses and refractive indices of all BCP films are summarized in Supplementary Table S1. From the data, a tendency towards an increasing refractive index with increasing sMWCNT content was observed. The refractive index of pure MWCNT would be higher than that of the block copolymer film (for comparison, the refractive index for a fullerene (C_60_) at 633 nm is *n* = 2.1). In addition, there is an influence of the pull-out speed during dip-coating, resulting in higher refractive index values for films prepared at a lower pull-out speed, possibly due to a higher tendency of MWCNT agglomeration, which is not completely understood yet.

**Figure 6:**
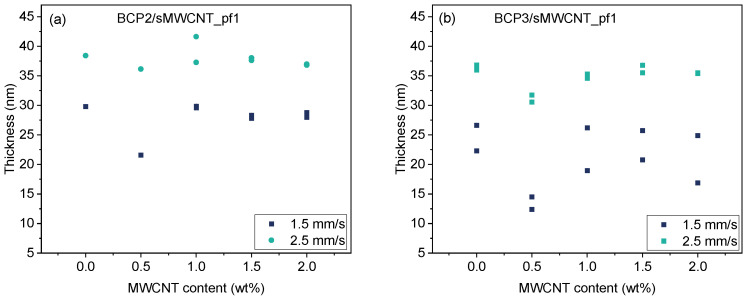
Film thickness of (**a**) BCP2 and (**b**) BCP3 as a function of MWCNT content at pull-out speeds of 1.5 mm s^−1^ (black) and 2.5 mm s^−1^ (cyan).

For BCP2 and BCP3, pull-out speeds of 1.5 mm s^−1^ and 2.5 mm s^−1^ were chosen to prepare thin films, as AFM and ellipsometry studies revealed that these pull-out speeds are sufficient to ensure complete wetting of the substrate, and thus the formation of homogeneous films. In Figure 6, the influence of MWCNT content on the film thickness of the BCP composites is plotted for varying pull-out speeds. Interestingly, a decrease in film thickness occurred for both BCPs and both pull-out speeds after addition of 0.5 wt% of sMWCNT_pf1, while further increasing MWCNT contents up to 2 wt% did not have any significant influence on the film thickness. It may be argued that addition of small amounts of MWCNT reduced the surface tension of the BCP solution significantly and reduced the wetting of the silica wafer, resulting in slightly reduced film thickness. Higher pull-out speeds obviously counterbalanced this effect.

Higher pull-out speed of 2.5 mm·s^−1^ resulted in higher film thicknesses of around 37 nm (BCP2/sMWCNT_pf1) and 35 nm (BCP3/sMWCNT_pf1), while a lower pull-out speed of 1.5 mm·s^−1^ gave film thicknesses of around 30 nm (BCP2/sMWCNT_pf1) and around 25 nm (BCP3/sMWCNT_pf1).

#### 3.4.2. Film Morphology Investigated by AFM in Phase Contrast

The morphology of thin BCP and BCP/MWCNT films obtained by dip-coating was first examined by AFM in phase contrast. It was assumed that larger phase angles will represent the harder PMMA phase while smaller phase angles result from the softer PsfMA phase. The average surface roughness (R_q_) of the thin films determined by AFM typically ranged between 1.7 and 3.5 nm (Supplementary Figure S2). This means that the signal contrast should not arise from differences in height but indeed from phase contrast.

**Figure 7:**
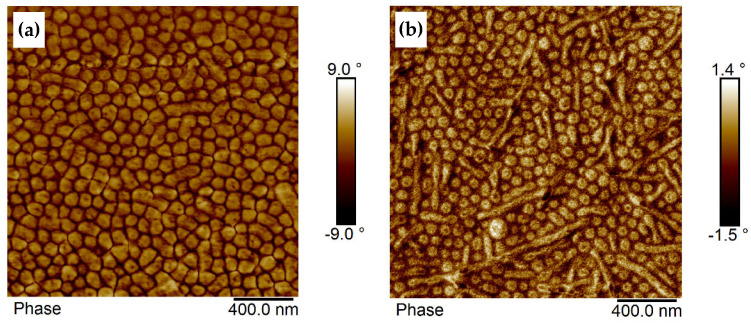
AFM phase images of (**a**) BCP1 film (thickness 32 nm) dip-coated at 1.5 mm/s and (**b**) BCP2 film (thickness 38 nm), dip-coated at 2.5 mm/s.

The morphologies of dip-coated films of BCP1 and BCP2, which are similar in their block composition and differ only in their molecular weight, having given film thicknesses of 32 nm and 38 nm, respectively, are illustrated in Figure 7a,b. Former investigations of thin films of BCP2 with film thicknesses below the d-spacing (distance between periodic structural features) in bulk performed by GISAXS and reported in [44] indicated a morphology with standing PMMA cylinders, as it was observed before in PMMA-b-P(pentylMA) diblock copolymer films [57]. It was assumed that the same morphology is formed in these thin films with film thickness below the bulk d-spacing.

The morphology of BCP1 is characterized by spherically arranged hard domains (PMMA) surrounded by a soft phase assigned to PsfMA (Figure 7a). The thin film of BCP2 with comparable film thickness of about 38 nm (again below the d-spacing in bulk) showed a mixture of hard spheres with a soft inner core plus lying cylinders (Figure 7b), whereas the bulk morphology was characterized by a mixture of cylinders and lamellae (Table 1 and [44]). Especially for BCP2 it is obvious that the hard PMMA domains exhibit a soft inner core that appears dark in the phase contrast. Thus, the thin film morphology is characterized by the semifluorinated phase (dark) positioned in the inner core of the hard spheres, a PMMA core layer and a PsfMA phase surrounding the PMMA phase. This morphology may result from micelle formation as proven before by cryo-TEM to occur in the THF solution [44].

The PMMA domain size and domain spacing (measured from domain center to domain center) of BCP1 films was ~82 nm and ~91 nm, respectively. This value is larger than the d-spacing in bulk found by SAXS (Table 1), which could be due to the micelle-like structure of the domains, containing a PsfMA-core.

**Figure 8:**
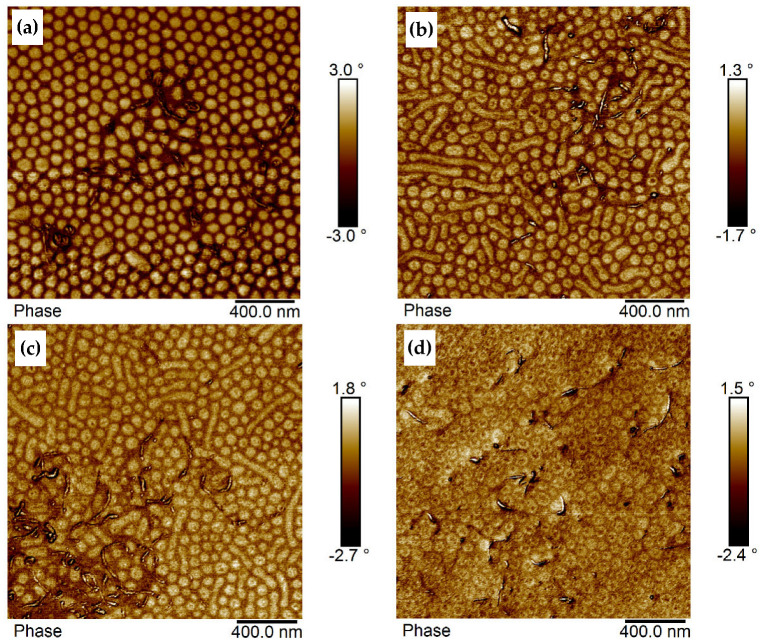
AFM phase images of (**a**) BCP1/0.5wt% sMWCNT_pf2 film dip-coated at 1.5 mm s^−1^, and BCP2 composite films with (**b**) 1 wt%, (**c**) 1.5 wt%, and (**d**) 2 wt% of sMWCNT_pf1, dip-coated at 2.5 mm s^−1^.

Upon introduction of modified sMWCNT into BCP1, attractive interactions occur between the perfluorinated sMWCNT and the semifluorinated side chains existing in each repeating unit of the PsfMA. The MWCNT are embedded in the soft phase and expanded it to some extent as shown in Figure 8a. The size of the PMMA domains was reduced from 82 nm to ~67 nm (Table 2). In fact, the addition of 0.5 wt% sMWCNT_pf2 yielded a more pronounced morphology.

The preferred localization of the modified sMWCNT into the soft (semifluorinated) phase, combined with the expansion of the embedding soft phase, was also exhibited in BCP2 films, as shown in Figure 8b,d, representing BCP2 based composite films with a filler content of 1 wt%, 1.5 wt% and 2 wt% sMWCNT_pf1. Figure 8c presents the phase image of a composite film with 1.5 wt% sMWCNTs_pf1. It exhibits a region in which the MWCNT are enriched, thus also showing an enrichment of the semifluorinated phase, demonstrating the strong interactions between the fluorine groups of the MWCNT with the semifluorinated side chains of the PsfMA block. Due to the expansion of the soft phase, the PMMA regions became smaller with increasing MWCNT content and transformed into spherical domains. This becomes particularly clear in Figure 8d, showing the morphology of a composite with 2 wt% sMWCNT_pf1. In this image the core–shell morphology with a soft core of the semifluorinated phase, a hard shell consisting of PMMA and a surrounding soft PsfMA phase is much more pronounced.

**Figure 9:**
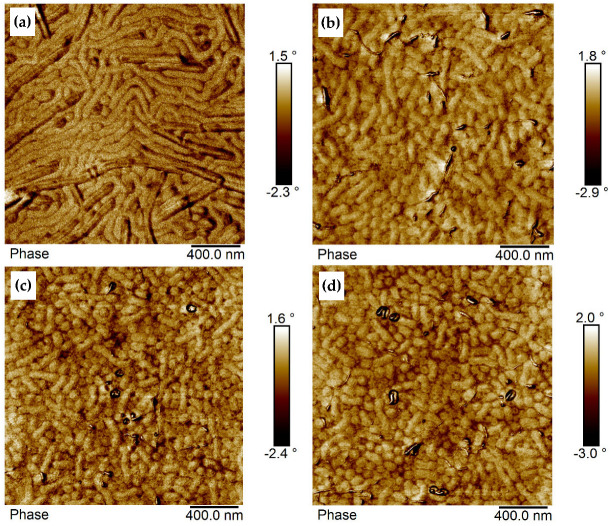
AFM phase images of (**a**) BCP3 and its composites with (**b**) 1 wt%, (**c**) 1.5 wt%, and (**d**) 2 wt% of sMWCNT_pf1, dip-coated at 2.5 mm s^−1^.

In BCP3 having a lamellar morphology in bulk [44], domains developed looking like lamellae and cylinders (Figure 9a). A quite similar morphology (cylinders with dark core surrounded by bright corona) was found by cryo-TEM of frozen THF solutions of BCP3 [44]. With increasing MWCNT content, the domains became shorter (Figure 9b). The addition of 0.5 to 1 wt% sMWCNT_pf1 resulted in the loss of the lamellar morphology and in the formation of cylinders or rods (Figure 9b). These rods were significantly shortened by further addition of MWCNT from 1.5 to 2 wt% (Figure 9c,d). The MWCNT preferentially accumulated in the semifluorinated phase which led to an interface curvature, and thus to the change in the morphology as also observed for the BCP2 (Figure 8). Such an influence of increasing CNT content on the self-assembly of block copolymers was previously found by Santos et al. [41] in solution-mixed SBS/MWCNT composites. The authors detected a significant decrease in long-range order of the domains when the volume fraction of MWCNT exceeded 1 vol%. Similar effects were reported earlier by Jang et al. [58] in poly(styrene-*b*-2-vinylpyridine) (PS-*b*-P2VP) diblock copolymers with modified gold particles localized in the spherical P2VP domain. With increasing nanoparticle fraction, the morphology changed to undoped P2VP cylinders. The alteration of the ordered morphology of poly(pentyl methacrylate-*b*-methyl methacrylate) diblocks by the addition of modified nanoparticles, such as gold, silver, magnetite and silica has also been discussed by Jehnichen et al. [23].

#### 3.4.3. Film Morphology Investigated by TEM and SEM

**Figure 10:**
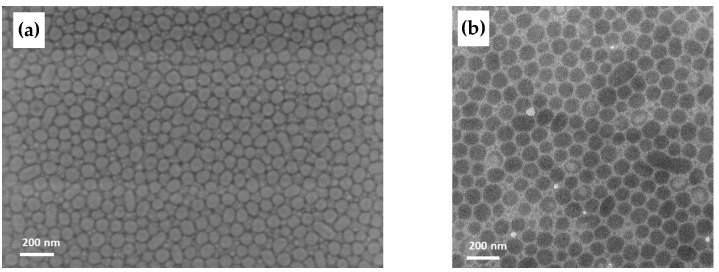
Images of a BCP2 thin film in (**a**) SEM and (**b**) TEM.

**Figure 11:**
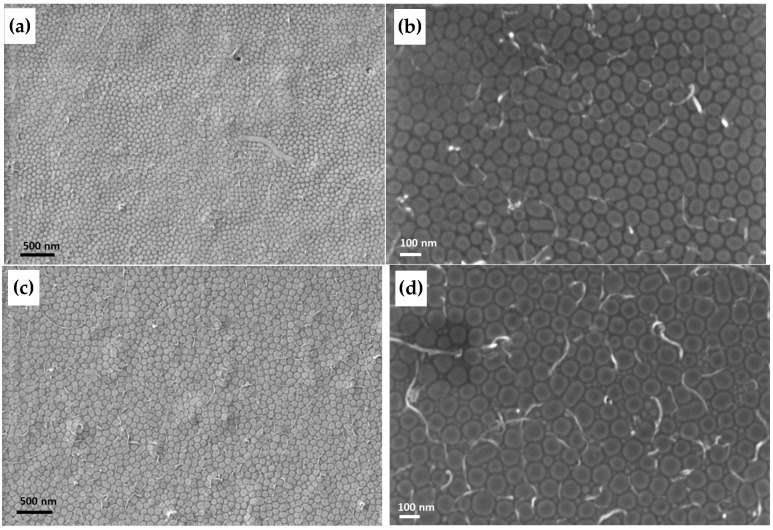
SEM images of BCP2 thin film containing 1 wt% unmodified sMWCNT using (**a**) secondary electrons detector and (**b**) InLens detector, and 1wt% modified sMWCNT_pf1 using (**c**) secondary electrons detector and (**d**) InLens detector.

A more detailed insight into the development of the thin film morphology was gained by structural investigation of BCP2 and BCP2/sMWCNT composites by SEM and TEM and energy-filtered TEM. BCP films prepared from THF solution and directly spread on a water surface clearly exhibited micelle formation with a PsfMA core and a PMMA shell as demonstrated in the Supplementary Materials and shown in Figure S3. This was observed before by cryo-TEM [44]. The morphology of a BCP2 film prepared by dip-coating as analyzed by SEM and TEM is shown in Figure 10a,b, respectively. SEM images of the morphology and MWCNT arrangement of BCP2 with unmodified and modified MWCNTs, respectively, are shown in Figure 11.

SEM and TEM images both appear to indicate the possibility that the micelles which were formed in solution of BCP2 and BCP2/sMWCNT (see Supplementary Figure S3), were transformed to the silicon wafer upon dip-coating, with the semifluorinated phase in the core and a PMMA shell. This was supported by elemental maps acquired by energy-filtered TEM of a BCP2 dipcoated film as shown in Supplementary Figure S4.

In the case of pure micelle formation, the sMWCNT have to be arranged between the micelles. In that case, the driving force of micelle formation is obviously greater than the driving force of the CNT to interact with the fluorinated BCP phase. However, the morphology change after addition of the sMWCNT as shown for BCP2 and BCP3 in Figure 8 and Figure 9 suggested a significant interaction of the perfluorinated sMWCNT with the semifluorinated phase. In addition, the low surface roughness measured by AFM also implies that the signal contrast arose from (soft–hard) phase contrast and not from height differences between individual micelles.

**Figure 12:**
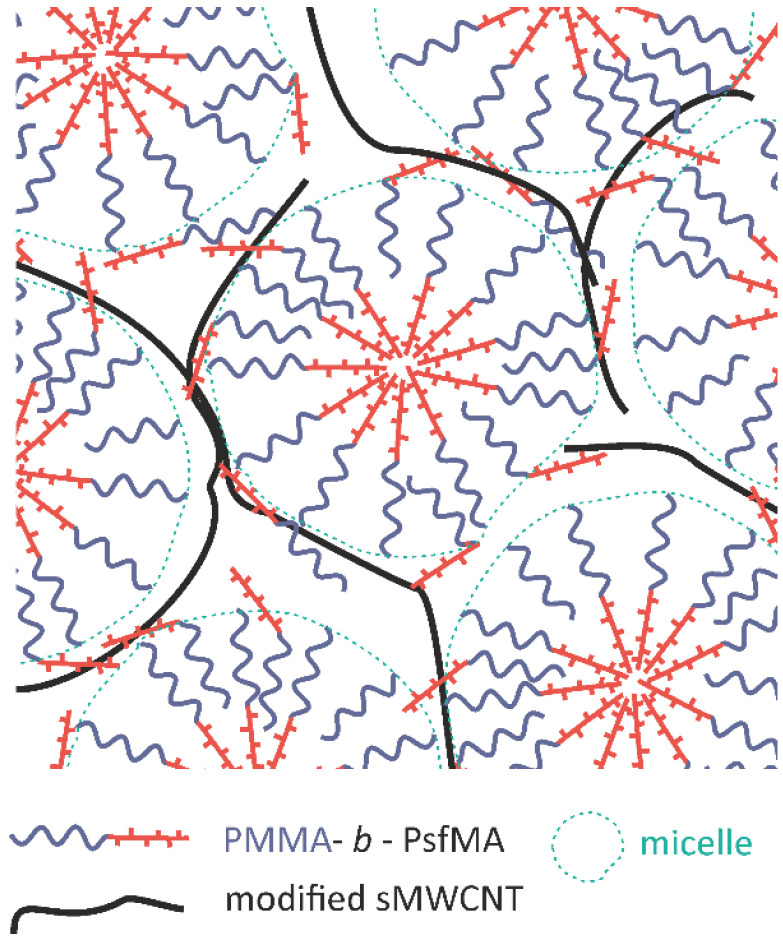
Scheme of the proposed PMMA-*b*-PsfMA BCP nanostructure in interaction with perfluor-modified sMWCNT.

A comparison of Figure 11a,c showed that the distances between the micelle-like domains appeared to be increased when modified sMWCNT were embedded (Figure 11c), as also observed in the AFM images (see Supplementary Figure S5d). However, the effect apparently did not occur when unmodified sMWCNT were embedded (Figure 11a). Comparative AFM studies on BCP2 with unmodified sMWCNT showed the same tendencies (Supplementary Figure S5b). These observations support our hypothesis that the perfluoroalkyl-modified sMWCNT interact more strongly with the semifluorinated phase of the BCP, leading to an enrichment of PsfMA chains in the phase surrounding the micelles, resulting in an improved dispersion of the MWCNTs. In addition, Figure 11b,d indicates that the perfluoralkyl-modified sMWCNT are almost entirely located between the micelle-like domains, while unmodified CNTs are positioned rather disordered both between the domains and overlapping them. The morphological BCP structure in interaction with the perfluoralkyl-modified sMWCNT is presented in a scheme in Figure 12.

#### 3.4.4. Chemical Composition of the Thin Films Surface Examined by XPS

**Figure 13:**
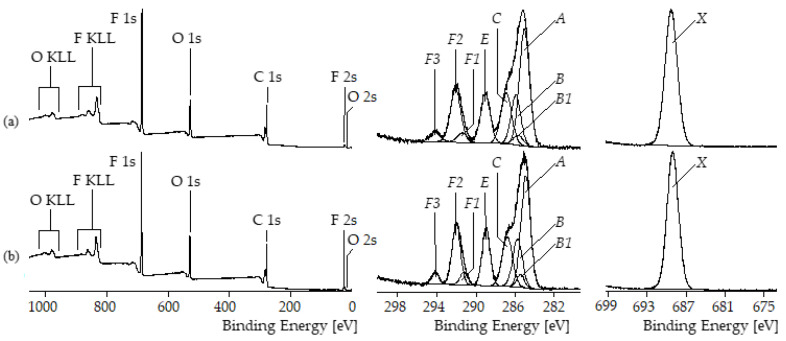
Wide-scan, C 1s and F 1s high-resolution XPS spectra recorded on (**a**) a pure BCP1 film, and (**b**) a BCP1 film containing sMWCNT_pf2.

The surface composition of the thin films based on BCP 1 was examined by XPS. According to the molecular structure of BCP1 as shown in Figure 4, the XPS spectra (Figure 13a, left column) showed the presence of carbon (as C 1s peak), oxygen (as O 1s, O 2s, and O KLL Auger series) and fluorine (as F 1s, F 2s, and F KLL Auger series). In a previous paper, we analyzed the shapes of the high-resolution C 1s spectra and assigned the component peaks to the chemical structure of the block copolymers [59]. The C 1s spectrum recorded from the sMWCNT_pf2-containing BCP1 film (Figure 13b, left column) does not differ from the C 1s spectrum of the pure BCP1 film. The relative fluorine content ([F]:[C]) is about 0.48 for both BCP films. In particular, no component peak (above referred to as Ph) showing photoelectrons from sp^2^-hybridized carbon atoms could be separated in the C 1s spectra (Figure 13b, middle column). Shake-up peaks were also not observed. Thus, photoelectrons from the embedded MWCNT do not contribute to the XPS spectra. The BCP completely encapsulates the MWCNT. The BCP films were thicker than the maximum information depth of the XPS method, which is about 8 nm. Thus, it can be concluded that the film surface is completely covered by the semifluorinated phase of the BCP. It was already found in a previous study that all BCP thin films were extremely hydrophobic, indicating an enrichment of the surface with fluorinated chains [44].

### 3.5. Electrical Conductivity of Nanocomposite Films

**Figure 14:**
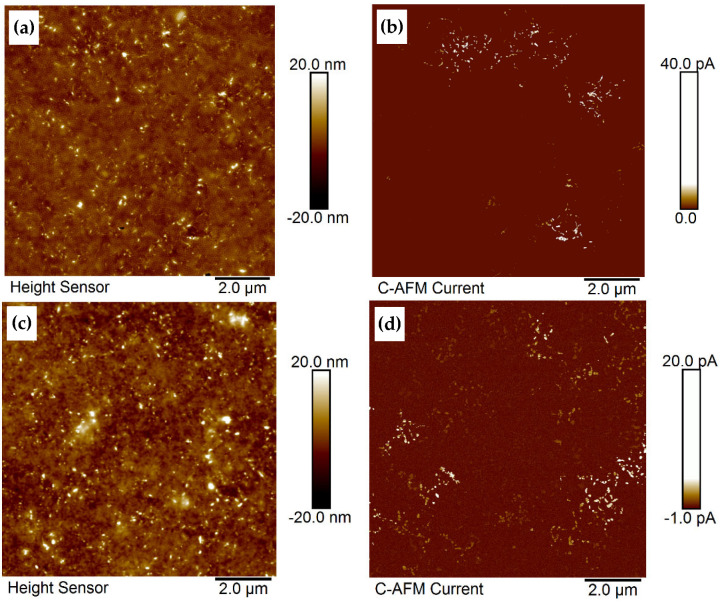
cAFM: (**a**,**c**) topography and (**b**,**d**) contact current signals of BCP2/2 wt% sMWCNT_pf1 composite film (thickness 37 nm) and BCP3/2 wt% sMWCNT_pf1 composite film (thickness 36 nm), respectively; dip-coated at 2.5 mm s^−1^, V = 400 mV.

For the electrical measurements by cAFM, the current was measured on the film layer or on the uncoated part of the wafer, respectively. No electrical current upon localization of the cAFM tip on the wafer surface, i.e., the silicon wafer surface is insulating, as expected. For the BCP2 and BCP3 composites, a conductive MWCNT network could be detected at a sMWCNT_pf1 concentration of 2 wt% in the polymer. For composite films with MWCNT contents below 2 wt% no current flow was detected. The electrical current flows from the lateral silver contacts through the CNT network and is visible as bright regions of local current flow in the AFM current images (Figure 14). In the EFM phase images (Supplementary Figure S6), the CNT network appears dark due to higher attractive electrostatic interaction with the AFM tip compared to the insulating polymer matrix. However, inhomogeneous regions are also detected in which the conductive MWCNT network is not generated or only partially generated. With increasing MWCNT content, the tendency to MWCNT agglomerate formation also increases.

**Figure 15:**
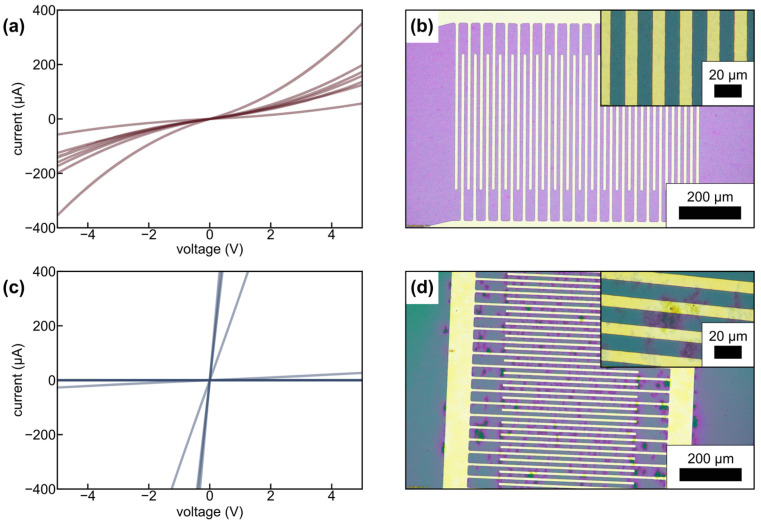
(**a**,**c**) Current-voltage characteristics measured using multiple interdigitated electrode pairs, covered with thin films of BCP2/MWCNT composites containing (**a**) 2 wt% perfluoroalkyl-modified and (**c**) 2 wt% non-modified sMWCNT, (**b**,**d**) respective optical micrographs of representative electrode pairs coated with BCP2/MWCNT composites containing (**b**) 2 wt% perfluoroalkyl-modified and (**d**) 2 wt% non-modified sMWCNT.

While cAFM gave qualitative insights on the MWCNT networks embedded in the BCP matrix materials, conductivity measurements were conducted on composite thin films of BCP2 with 2 wt% of sMWCNT_pf1 deposited onto lithographically fabricated microelectrode structures. Therefore, interdigitated electrode microstructures (spacing of 10 µm) were fabricated and coated with composites of modified and non-modified sMWCNT. Figure 15a,c show the current-voltage characteristics of BCP2/sMWCNT film samples fabricated with 2 wt% perfluoroalkyl-modified and 2 wt% non-modified sMWCNT, respectively. Upon biasing the electrodes with voltages between −5 and 5V, films from BCP2 containing perfluoroalkyl-modified sMWCNT showed reproducible non-ohmic behavior with currents in the 100 µA range (Figure 15a). In contrast, samples from BCP2 with non-modified sMWCNT exhibited either extremely low conduction with currents in the pA range or pronounced conductivity on different electrode pairs (Figure 15c). Hence, the conductance of electrode structures coated with BCP2/sMWCNT_pf1 showed significantly lower scattering than the conductance of their counterparts coated with BCP2/sMWCNT. The still non-negligible variations between electrode pairs were attributed to a somewhat inhomogeneous film thickness of the samples (see Supplementary Table S2, samples A1–A3).

Figure 15b,d depicts representative micrographs of interdigitated electrodes covered with the respective BCP composite films. While the films fabricated from BCP2 containing 2 wt% perfluoroalkyl-modified sMWCNT showed a homogeneous MWCNT dispersion, agglomerates of MWCNT located between the interdigitated electrodes of some electrode pairs are clearly visible for the sample fabricated from BCP2 with 2 wt% of non-modified sMWCNT. These differences in the MWCNT dispersion are also visible in corresponding SEM images (see Supplementary Figure S7). Such inhomogeneities, attributed to poor dispersibility of non-modified sMWCNT in the BCP matrix, presumably led to low impedance pathways connecting some electrode pairs and causing high currents for some individual devices, while no significant current flow was observed for others.

Due to the strongly hydrophobic nature of the perfluoroalkyl groups the van der Waals attraction between the MWCNT most likely decreases and can lead to a better dispersion of the sMWCNT_pf1 compared to the non-modified sMWCNT, which have a higher tendency to agglomerate. Furthermore, as already discussed, the interaction between the perfluoralkyl-modified sMWCNT and the fluorinated side chains of the BCP led to a more homogeneous distribution and dispersion of the modified sMWCNT in the polymer matrix. As a result, a homogeneous, reliable electrical conductivity is achieved within the composite in contrast to films with unmodified MWCNTs.

The presence of functional groups, such as oxygenated groups can also affect the electrical properties of MWCNT and thereby reduce the electrical conductivity of the composites [60]. In the present study, however, only a small number of C-OH groups were introduced onto the surface of the MWCNT by grinding. An [O]:[C] ratio of only 0.031 was observed (i.e., approximately three out of every 100 carbon atoms are bonded to an oxygen atom; see Section 3.1). Their influence on the conductivity was therefore considered negligible. Larger changes in the electrical conductivity are to be expected due to the grafting of the perfluorinated compounds, which can have an insulating effect [61]. The overall effect on the electrical conductivity results from a balance between better distribution of modified sMWCNT and possible insulating effects of the fluorine-containing groups. The electrical conductivity of the BCP2/sMWCNT composite containing 2 wt% of perfluoroalkyl-modified sMWCNT was calculated based on slope fits to the low-voltage range (−1 to 1 V) of the current-voltage data shown in Figure 15a. A value of $\sigma =9\cdot 10^{-4}\;S\;{\mathrm{cm}}^{-1}$ was determined, which is in the typical range of the conductivity of PMMA/CNT bulk nanocomposites [9,62]. The formation of a conductive network in polymer/CNT composites can be explained by classical percolation theory [63,64]. A sharp increase in electrical conductivity occurs once the critical filler content is exceeded. Due to the limited number of characterized compositions, the electrical percolation threshold of the BCP composite films could not be determined. However, based on the C-AFM studies, it is assumed to be between 1.5 and 2 wt% MWCNT content. In an earlier study it was shown that the percolation threshold of SB based BCP composites increased successively by shortening the CNT [39]. Investigation of the percolation behavior of CNTs in block copolymers will be subject of further research.

## 4. Conclusions

The nanosized morphologies of PMMA-b-PsfMA diblock copolymers were intended to be used as matrices for the selective localization of carbon nanotubes to develop functional polymer composites with defined and reproducible electrical transport behavior. To facilitate the incorporation of the nanotubes into the block copolymer nanodomains, short sMWCNT with a length of ~500 nm were employed. Additionally, the sMWCNT were modified with perfluoroalkyl groups to enhance the compatibility of the semifluorinated phase of the BCP with the sMWCNT. The modified sMWCNT were dispersed in THF via ultrasonic treatment. Thin polymer composite films were prepared by solution mixing of the dispersed sMWCNT with the polymer solution followed by dip-coating the final mixture onto silicon substrates. The film thicknesses depended on the pull-out speed used in the dip-coating process and varied between 30 and 60 nm. The resulting morphologies of the BCP in bulk depended on the block composition and molecular weight and varied between spheres, cylinders and lamellae. SEM and TEM investigations indicated that the block copolymers formed micelles in the thin films with a PsfMA core and a PMMA shell as opposed to the morphologies expected from former SAXS and GISAXS measurements. Micelle formation was observed in THF solution and obviously dominated the morphology development in dip-coated films. AFM studies suggested that a soft PsfMA phase in which the sMWCNT are localized was generated around the core–shell micelles due to compression of the micelle structure upon dip-coating. The dispersion of the sMWCNT was significantly more effective when they were modified with perfluoroalkyl groups, which also indicated that they interacted more intensively with the fluorinated BCP phase. This resulted in the formation of an ordered, electrically conductive network. Thus, thin films of BCP with perfluorinated sMWCNT proved to exhibit reliable electrical conductivity. Applications for such nanocomposites with well-dispersed CNTs can include gas and liquid sensing. The self-assembly of CNTs is also very interesting for applications in microelectronics.

## Figures and Tables

**Table 1:** Chemical characteristics of the used PMMA-*b*-PsfMA diblock copolymers (bulk samples), from [44,47,48].

BCP Sample	Composition MMA/sfMA (mol mol^−1^)	*M_n_* (kg mol^−1^)	*Đ* = *M_w_*/*M_n_*	Morphology in Bulk (TEM, SAXS)	d-Spacing (SAXS) (nm)
BCP1 (PMF187)	73.5/26.5	22.0	1.18	Mixture of lamellae and cylinders	41 (CYL/LAM) [48]
BCP2 (PMF102)	73/27	26.2	1.10	Mixture of lamellae and cylinders	54 (CYL/LAM) [44]
BCP3 (PMF106)	54/46	18.8	1.06	Lamellae	45 (LAM) [44,47]

**Table 2:** Morphological characteristics of thin films prepared from BCP1 and its composite with 0.5 wt% sMWCNT.

BCP Sample	Film Thickness ^1^ (nm)	Morphology (AFM)	PMMA Domain Size ^2^ (nm)	Periodic Nanostructure ^3^ (nm)
BCP1	32	spherical	82	91
BCP1/0.5 wt% sMWCNT_pf2	31	spherical	67	91

^1^ spectroscopic ellipsometry, ^2^ measured on AFM images; ^3^ measured on AFM images from domain center to domain center.

## Data Availability

Data generated during the study are archived on the research institute’s internal server and can be provided by the corresponding author upon reasonable request.

## Supplementary Materials

The following supporting information can be downloaded at: https://www.mdpi.com/article/10.3390/polym17091271/s1, Table S1: Optical layer parameters (thickness, refractive index) and error of fit (MSE) from modeling of ellipsometric data of BCP and composite thin films; Table S2: Average film thickness of BCP2/sMWCNT thin films measured via ellipsometry and topographic AFM; Figure S1: Representative topographic AFM image from sample A2 of a thin film from BCP/MWCNT composites containing perfluoroalkyl-modified sMWCNT recorded at the film edge; Figure S2: AFM height images of (a) BCP1 film (Rq: 2.32 nm) dip-coated at 1.5 mm/s and (b) BCP2 film (Rq: 1.74 nm), dip-coated at 2.5 mm/s; Figure S3: Bright field TEM image and corresponding elemental maps acquired by energy-filtered TEM of the micelles of BCP2 prepared from THF solution spread onto water surface: Fluorine mapping detects PsfMA in the core of the micelles; Figure S4: Bright field TEM image and corresponding elemental maps acquired by energy-filtered TEM of BCP2 dipcoated film; Figure S5: AFM investigation of BCP2 dipcoated films with (a) and (b) 1 wt% unmodified sMWCNT and (c) and (d) perfluoralkyl-modified sMWCNT_pf1, height and phase images, respectively; Figure S6: Electrical force gradient microscopy (EFM): (a) and (c) topography and (b) and (c) phase image of BCP2/2 wt% sMWCNT_pf1 composite film (thickness 37 nm) and BCP3/2 wt% sMWCNT_pf1 composite film (thickness 36 nm), respectively; prepared from THF, dip-coated at 2.5 mm s-1: The CNT network appears dark in the phase images due to higher attractive electrostatic interaction with the AFM tip compared to the insulating polymer matrix; Figure S7: SEM images of electrode pairs dip-coated with BCP2 composite thin films containing (a) and (b) 2 wt% unmodified sMWCNT, (c) and (d) 2wt% modified sMWCNT_pf1 using InLens detector: agglomerated structure of unmodified sMWCNTs compared to well dispersed modified sMWCNT_pf1.
