# Supplementary material for: MWCNT Localization and Electrical Percolation in Thin Films of Semifluorinated PMMA Block Copolymers
Source: Polymers (Basel). 2025 May 6;17(9):1271. doi: 10.3390/polym17091271 (PMC12073811; doi:10.3390/polym17091271)
Supplement: Supplementary file 1 [file polymers-17-01271-s001.zip › polymers-3610358-supplementary.pdf]

Supplementary Materials

MWCNT Localization and Electrical Percolation in Thin Films of Semifluorinated PMMA Block Copolymers

Ulrike Staudinger *, Andreas Janke, Frank Simon, Lothar Jakisch, Eva Bittrich, Petr Formanek, Lukas Mielke, Hendrik Schlicke, Qiong Li, Kathrin Eckstein and Doris Pospiech

Leibniz-Institut für Polymerforschung Dresden e.V., Hohe Str. 6, 01069 Dresden, Germany
* Correspondence: staudinger@ipfdd.de

Table S1. Optical layer parameters (thickness, refractive index) and error of fit (MSE) from modeling of ellipsometric data of BCP and composite thin films.

Sample	Code	MWCNT content (wt%)	Pull-out speed (mm/s)	Thickness (nm)	Deviation (nm)	MSE	Refractive index (633nm)	Comments
PMF187	1051	-	0.5				-	no evaluation possible, layer too inhomogeneous
	1052	-	0.5				-	no evaluation possible, layer too inhomogeneous
	1061	-	1.5	32.10	0.03	0.975	1.413	
	1062	-	1.5	32.18	0.03	1.030	1.407	
	1071	-	2.5	39.95	0.02	0.840	1.408	
	1072	-	2.5	38.57	0.02	0.789	1.412	
	1081	-	5.0	62.29	0.03	1.698	1.396	
	1082	-	5.0	58.67	0.02	1.193	1.402	
	1111	0.5	1.5	29.93	0.06	0.918	1.425	
	1112	0.5	1.5	31.78	0.06	0.848	1.419	
	1121	0.5	2.5	42.52	0.04	0.399	1.412	
	1122	0.5	2.5	43.68	0.04	0.294	1.419	
	1131	0.5	5.0	62.67	0.06	0.524	1.415	
	1132	0.5	5.0	57.41	0.05	0.467	1.413	
PMF102	1911	-	1.5	29.82	0.06	0.884	1.430	
	1921	-	2.5	38.42	0.05	0.794	1.426	
	1931	0.5	1.5	21.58	0.21	4.905	1.496	refitted, still n too high
	1941	0.5	2.5	36.14	0.05	0.807	1.425	
	1951	1.0	1.5	29.61	0.06	0.957	1.448	
	1952	1.0	1.5	29.87	0.06	0.953	1.446	
	1961	1.0	2.5	37.26	0.05	0.846	1.435	
	1962	1.0	2.5	41.62	0.04	0.714	1.430	
	1971	1.5	1.5	27.81	0.07	1.098	1.479	
	1972	1.5	1.5	28.34	0.07	1.072	1.471	
	1981	1.5	2.5	37.61	0.05	0.796	1.444	

	1982	1.5	2.5	38.04	0.05	0.886	1.451	
	1991	2.0	1.5	28.77	0.07	1.268	1.497	
	1992	2.0	1.5	27.97	0.09	1.629	1.506	
	19101	2.0	2.5	36.98	0.06	0.983	1.47	
	19102	2.0	2.5	36.8	0.06	1.017	1.469	
PMF106	2111	-	1.5	26.64	0.27	4.973	1.522	
	2112	-	1.5	22.29	0.38	9.115	1.613	
	2121	-	2.5	36.82	0.14	2.127	1.429	
	2122	-	2.5	36.00	0.09	1.356	1.422	
	2131	0.5	1.5	12.39	0.18	12.817	-	refitted, but n not reasonable
	2132	0.5	1.5	14.51	0.27	12.009	-	refitted, but n not reasonable
	2141	0.5	2.5	30.58	0.07	0.956	1.425	
	2142	0.5	2.5	31.76	0.06	0.918	1.426	
	2151	1.0	1.5	18.98	0.13	2.447	1.576	
	2152	1.0	1.5	26.20	0.08	1.114	1.448	
	2161	1.0	2.5	35.30	0.06	0.984	1.44	
	2162	1.0	2.5	34.59	0.06	0.981	1.437	
	2171	1.5	1.5	20.77	0.10	1.390	1.499	
	2172	1.5	1.5	25.75	0.08	1.144	1.465	
	2181	1.5	2.5	36.80	0.06	0.949	1.445	
	2182	1.5	2.5	35.52	0.06	1.013	1.447	
	2191	2.0	1.5	16.88	0.18	4.179	1.641	refitted
	2192	2.0	1.5	24.89	0.08	1.274	1.489	
	21101	2.0	2.5	35.41	0.06	1.033	1.457	
	21102	2.0	2.5	35.51	0.07	1.049	1.454	

Film thickness evaluation for BCP2/sMWCNT thin films dip-coated on electrode structures

To determine the samples' film thickness for computation of their conductivity, topographic AFM measurements were carried out at the edges of scratches manually introduced into the composite films, in vicinity of the electrode structures. An exemplary AFM scan depicted in **Figure S1** clearly shows the film (elevated) and substrate section (lower). Recorded data were analyzed using the software Gwyddion 2.60. After leveling by taking into account height data recorded on the substrate section, terraces were fit to both sections using Gwyddion's built-in "terraces" function. The film thickness t was then determined from the height difference between the terraces for each scan. Values denoted in **Table S2** represent the average film thickness determined from at least three AFM scans at different positions for each sample.

Table S2. Average film thickness of BCP2/sMWCNT thin films measured via ellipsometry and topographic AFM.

Sample	Ellipsometry (t in nm)	AFM ($t \pm \sigma$ in nm)
A1	-	86 ± 21
A2	-	93 ± 11
A3	-	106 ± 2
A4*	98	-
B1	-	87 ± 20
B2	-	80 ± 2
B3*	75	-

*The sample did not contain electrode structures

AFM measurements were conducted on three film samples (A1-A3) from BCP2 fabricated using per-fluoroalkyl-modified sMWCNT and two film samples (B1-B2) from BCP2 fabricated using non-modified sMWCNT. It should be noted that the films prepared on electrode structures for electrical conductivity measurements exhibited a greater thickness compared to the other samples reported in the study (see **Table S1**). This is attributed to the reuse of a BCP2/2 wt% sMWCNT_pf1 solution that had been stored for few weeks, during which minor evaporation of the solvent occurred, resulting in a slightly increased polymer concentration. **Figure S1** shows a representative AFM scan recorded on sample A2. For samples A1 to A3, the film thickness ranged between 86 nm and 106 nm, while samples B1 and B2 showed a film thickness of 87 nm and 80 nm, respectively. Corresponding film thickness values measured by means of ellipsometry for composite films containing the modified and non-modified sMWCNT are ~98 nm and ~75 nm, respectively. Samples analyzed by means of ellipsometry were prepared analogously to samples used for conductivity/AFM measurements. However, their susbtrates did not contain electrode structures.

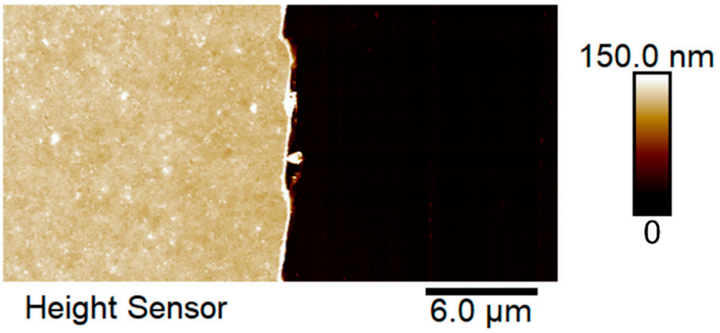

Figure S1. Representative topographic AFM image from sample A2 of a thin film from BCP/MWCNT composites containing perfluoroalkyl-modified sMWCNT recorded at the film edge.

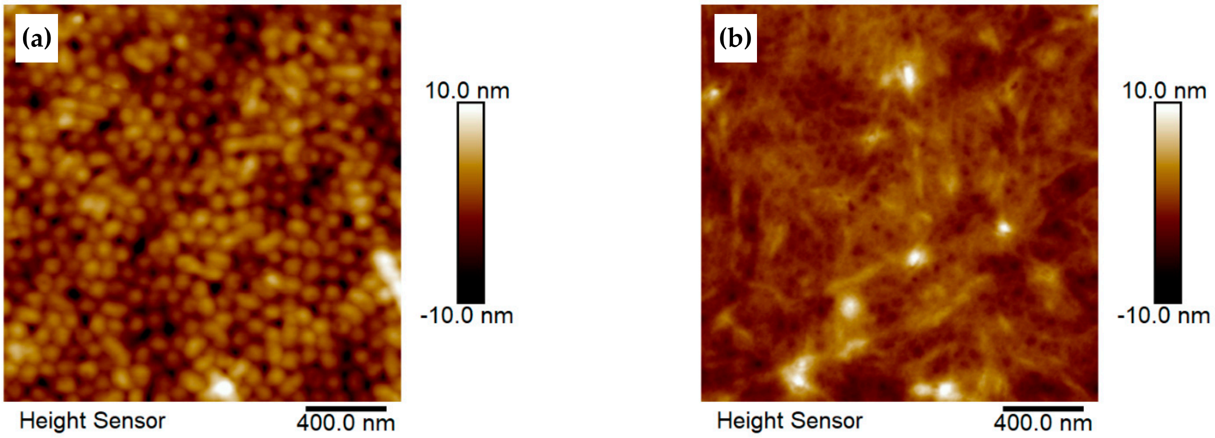

Figure S2. AFM height images of (a) BCP1 film (Rq: 2.32 nm) dip-coated at 1.5 mm/s and (b) BCP2 film (Rq: 1.74 nm) , dip-coated at 2.5 mm/s.

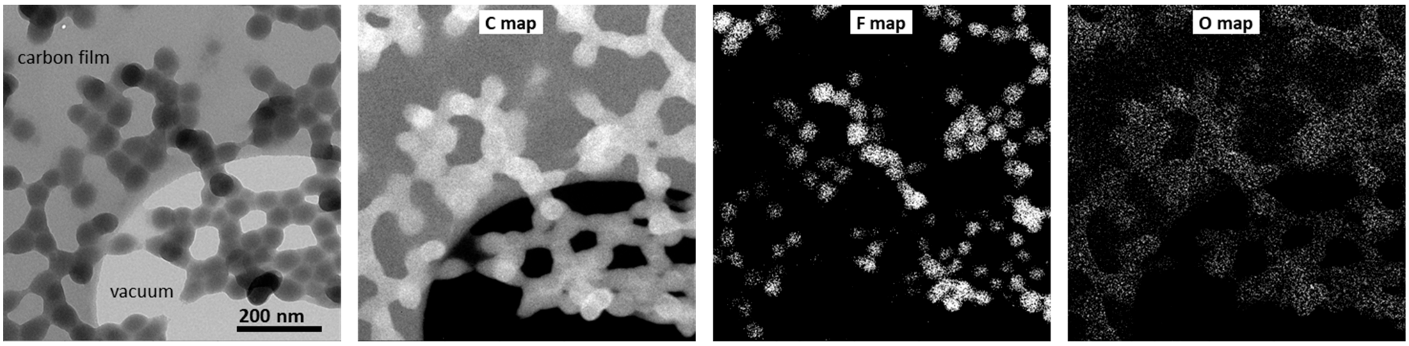

Figure S3. Bright field TEM image and corresponding elemental maps acquired by energy-filtered TEM of the micelles of BCP2 prepared from THF solution spread onto water surface: Fluorine mapping detects PsfMA in the core of the micelles.

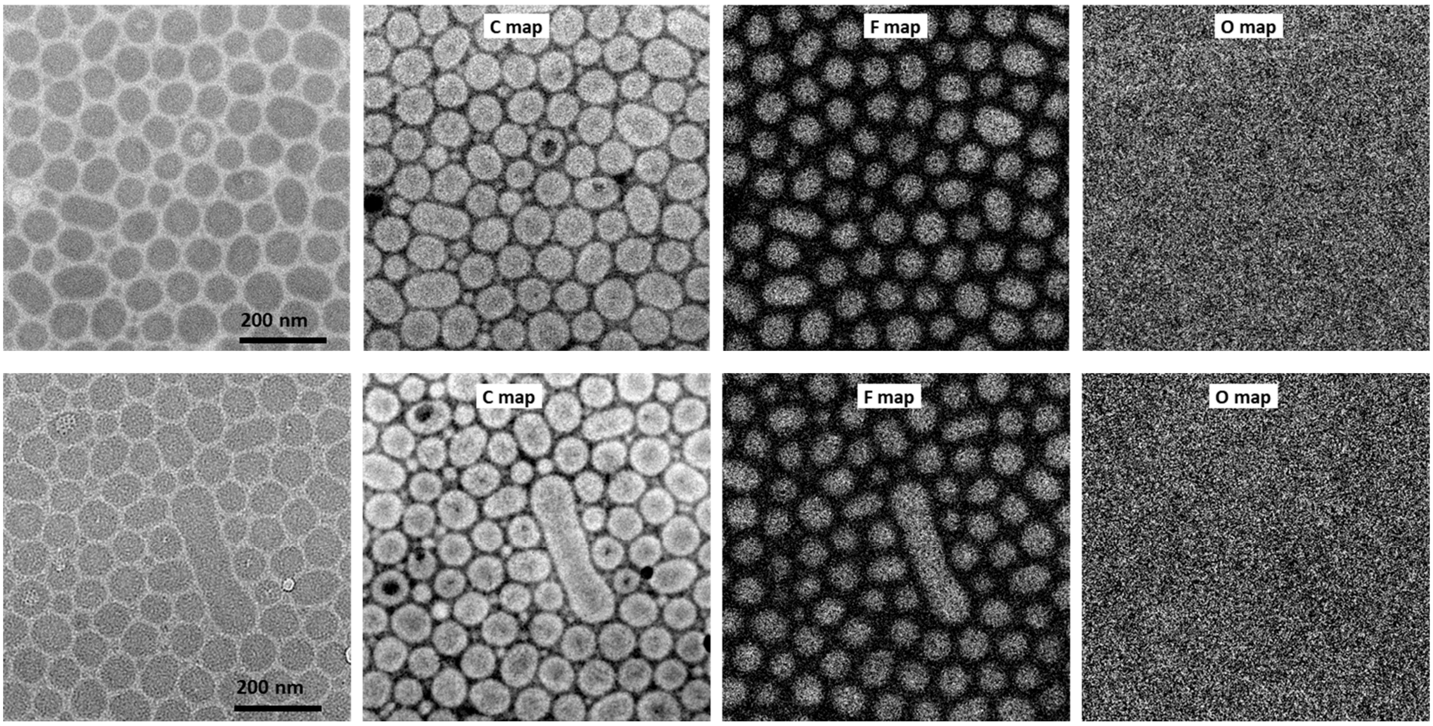

Figure S4. Bright field TEM image and corresponding elemental maps acquired by energy-filtered TEM of BCP2 dipcoated film.

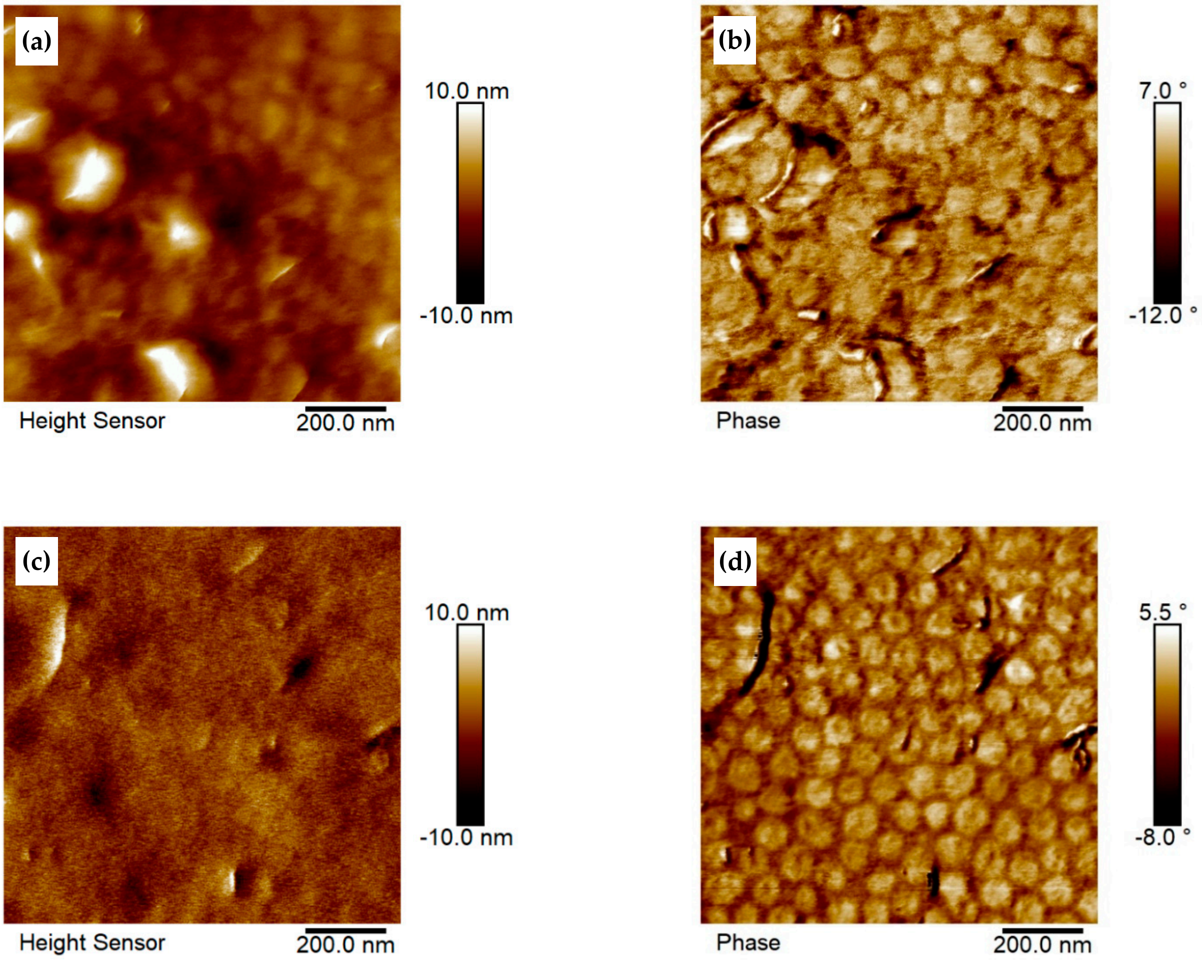

Figure S5. AFM investigation of BCP2 dipcoated films with (a) and (b) 1 wt% unmodified sMWCNT and (c) and (d) perfluoralkyl-modified sMWCNT_pf1, height and phase images, respectively.

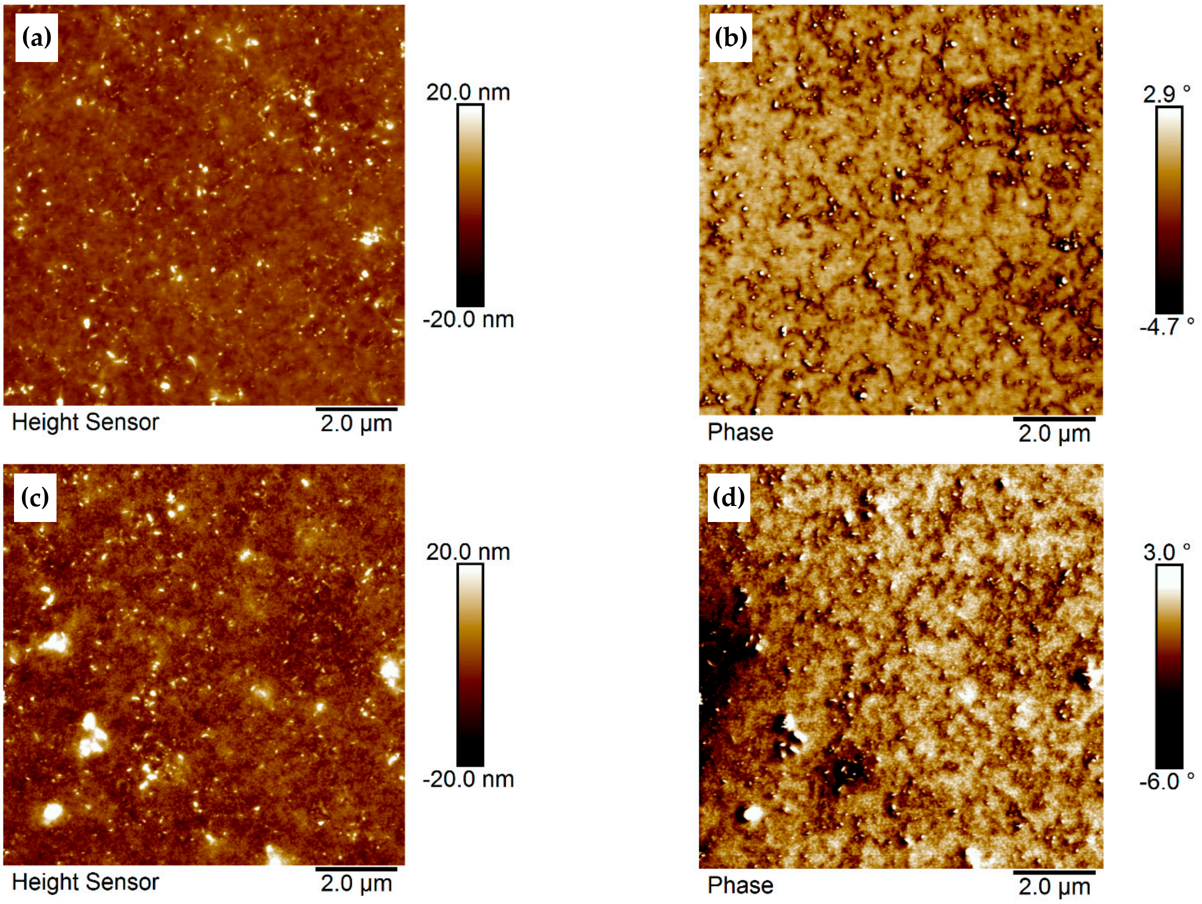

Figure S6. Electrical force gradient microscopy (EFM): (a) and (c) topography and (b) and (c) phase image of BCP2/2 wt% sMWCNT_pf1 composite film (thickness 37 nm) and BCP3/2 wt% sMWCNT_pf1 composite film (thickness 36 nm), respectively; prepared from THF, dip-coated at 2.5 mm s-1: The CNT network appears dark in the phase images due to higher attractive electrostatic interaction with the AFM tip compared to the insulating polymer matrix.

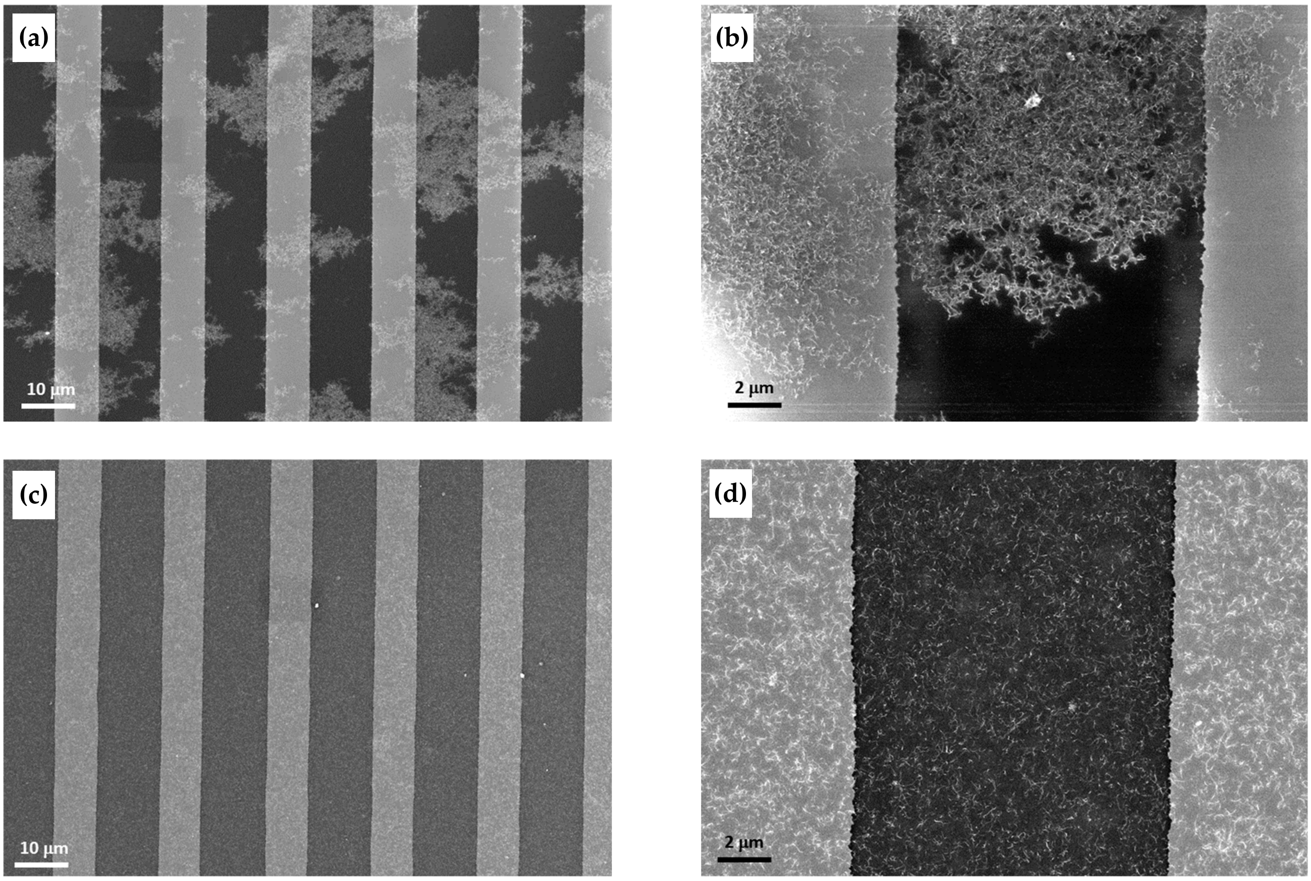

Figure S7. SEM images of electrode pairs dip-coated with BCP2 composite thin films containing (a) and (b) 2 wt% unmodified sMWCNT, (c) and (d) 2wt% modified sMWCNT_pf1 using InLens detector: agglomerated structure of unmodified sMWCNTs compared to well dispersed modified sMWCNT_pf1.
